# Comparison of neuropsychological profiles in children and adolescent with anorexia nervosa and avoidant/restrictive food intake disorder (ARFID)

**DOI:** 10.1192/j.eurpsy.2021.942

**Published:** 2021-08-13

**Authors:** C. Basile, F. Gigliotti, M. Colaiori, F. Di Santo, A. Terrinoni, I. Ardizzone, U. Sabatello

**Affiliations:** 1 Department Of Human Neuroscience,section Of Child And Adolescent Neuropsychiatry, Sapienza University of Rome, Roma, Italy; 2 Department Of Human Neuroscience, Section Of Child And Adolescent Neuropsychiatry, Sapienza University of Rome, Roma, Italy; 3 Department Of Human Neuroscience, Sapienza University of Rome, Section of Child and Adolescent Neuropsychiatry, RM, Italy; 4 Department Of Human Neuroscience, Sapienza University of Rome, Roma, Italy

**Keywords:** Avoidant/Restrictive Food Intake Disorder, anorexia nervosa, neurodevelopment, executive function

## Abstract

**Introduction:**

Anorexia Nervosa (AN) is an eating disorder characterized by low body weight, fear of gaining weight and distorted perception of body. Patients have rigidity, repetition of thoughts, alterations in decision-making skills and poor ability to provide new solutions. Avoidant/Restrictive Food Intake Disorder (ARFID) is a new eating disorder characterized by the absence of distress about body shape or fear of weight gain. Studies on neurocognitive aspects are few and no effective treatments are known.

**Objectives:**

The aim of our study was to further investigate the executive functions’ domains in AN and ARFID children and adolescents, to provide possible distinct neurocognitive traits in these patients.

**Methods:**

AN or ARFID patients (15 + 15; range 6-18 years), were assessed by neuropsychological tools, such as: Wechsler Intelligence Scale to measure I.Q. profile, NEPSY-II to explore attention and executive functions, Tower of London test to detect planning and problem solving abilities, the Bells Test to evaluate visual selective and focused attention, the Wisconsing Card Sorting Test (WCST) for assessment of flexibility and directing behaviors by achieving a goal and the Rey-Osterrieth complex figure test (ROCF) to assess visual-spatial abilities.

**Results:**

Patients with ARFID presented impairments in several executive functions domains, with difficulties in the impulse inhibition, in the sustained attention and in visual-spatial skills. Finally, in their anamnesis a higher comorbidity with neurodevelopmental disorders such as specific learning disorder has been underlined.

**Conclusions:**

The identification of specific deficit in neuropsychological profile of ARFID patients could be a rehabilitation target, together with standardized treatment.

